# Abundance and genetic damage of barn swallows from Fukushima

**DOI:** 10.1038/srep09432

**Published:** 2015-04-02

**Authors:** A. Bonisoli-Alquati, K. Koyama, D. J. Tedeschi, W. Kitamura, H. Sukuzi, S. Ostermiller, E. Arai, A. P. Møller, T. A. Mousseau

**Affiliations:** 1 Department of Biological Sciences, University of South Carolina, Columbia, SC 29208, USA; 2 Japan Bird Research Association, Fuchu, Tokyo, Japan; 3 Department of Physics and Astronomy, University of South Carolina, Columbia, SC 29208, USA; 4 Faculty of Environmental Studies, Tokyo City University, Yokohama City, Japan; 5 Value Frontier Co., Ltd., Minato, Tokyo, Japan; 6 Division of Ecology and Evolutionary Biology, Graduate School of Life Sciences, Tohoku University, Sendai, Japan; 7 Laboratoire d'Ecologie, Systématique et Evolution, CNRS UMR 8079, Université Paris-Sud, Bâtiment 362, F-91405 Orsay Cedex, France

## Abstract

A number of studies have assessed or modeled the distribution of the radionuclides released by the accident at the Fukushima-Daiichi Nuclear Power Plant (FDNPP). Few studies however have investigated its consequences for the local biota. We tested whether exposure of barn swallow (*Hirundo rustica*) nestlings to low dose ionizing radiation increased genetic damage to their peripheral erythrocytes. We estimated external radiation exposure by using thermoluminescent dosimeters, and by measuring radioactivity of the nest material. We then assessed DNA damage by means of the neutral comet assay. In addition, we conducted standard point-count censuses of barn swallows across environmental radiation levels, and estimated their abundance and local age ratio. Radioactivity of nest samples was in the range 479–143,349 Bq kg^−1^, while external exposure varied between 0.15 and 4.9 mGy. Exposure to radioactive contamination did not correlate with higher genetic damage in nestlings. However, at higher levels of radioactive contamination the number of barn swallows declined and the fraction of juveniles decreased, indicating lower survival and lower reproduction and/or fledging rate. Thus, genetic damage to nestlings does not explain the decline of barn swallows in contaminated areas, and a proximate mechanism for the demographic effects documented here remains to be clarified.

On March 11 2011, a tsunami caused by the Great East Japan Earthquake seriously damaged the electrical and the cooling systems of the Fukushima-Daiichi Nuclear Power Plant (FDNPP), causing hydrogen explosions at the Unit 1, 2 and 3 reactors. These explosions released large amounts of high volatility fission products, including ^129m^Te, ^131^I, ^133^Xe, ^134^Cs, ^136^Cs, and ^137^Cs^1^,^2^,^3^. Although the estimates of the release vary considerably^4^,^5^,^6^,^7^,^8^,^9^,^10^, the accident is universally regarded as the second largest release of radionuclides in history after the Chernobyl accident, with estimates of total radioactivity released in the range of up to hundreds of PBq^10^. Such massive discharge of radionuclides raises concern about its possible consequences for environmental and human health^11^, particularly given the persistence of ^137^Cs in the environment.

Predictably, large efforts have since been devoted to model the atmospheric release, the deposition of radionuclides and their redistribution^2^,^12^. Several other studies have assessed the concentration of the radionuclides in the biological tissues of animals and plants (mammals:^13^,^14^; fish:^7^,^15^; birds:^16^; plants^17^,^18^,^19^).

Few studies so far have examined the potential biological consequences of exposure to radionuclides released by the accident. A study on the pale blue grass butterfly (*Zizeeria maha*) that coupled field sampling and rearing of individuals under common garden conditions showed an increase in aberrations in the coloration and patterns of wings^20^,^21^. A study on earthworms also demonstrated that animals from sites where radiation level was as low as 2.8 μSv/h had higher DNA damage than animals from control sites^22^. A recent study of wild Japanese macaques (*Macaca fuscata*) found that individuals from Fukushima had lower white blood cell (WBCs) and red blood cell counts (RBCs), lower hemoglobin concentration and lower hematocrit values than those sampled in the Shimokita peninsula, at a distance of 400 km from the FDNPP^23^. Vitamin A levels of streaked shearwaters (*Calonectris leucomelas*) sampled in colonies exposed to contamination from the FDNPP were lower than in animals from a colony that was not reached by the plume^24^.

Ecological studies conducted in the Chernobyl Exclusion Zone have indicated that radiation levels comparable to those found around Fukushima can be associated with deleterious genetic, physiological and life-history consequences for exposed wildlife^25^. Low-dose radiation in the Chernobyl region was associated with higher DNA damage in adult barn swallows^26^, higher frequency of morphological abnormalities and tumors^27^,^28^, and a reduction in brain size^29^. These and other physiological and genetic consequences of radiation exposure in Chernobyl^30^ have been indicated as the likely cause underlying the higher mortality and the populations declines of many bird species living in the Chernobyl region, as inferred from point count censuses^31^ and age ratios from mist netting studies^32^.

In spite of differences between the two accidents in the quality and amount of contaminants scattered and the number of generations of exposure, early studies suggest that similarities also exist in the response of natural populations to radioactive contamination. Point-count censuses conducted around Fukushima in 2011 have found that bird population in radioactively contaminated areas have declined similarly as in Chernobyl^33^. Later surveys validated this finding and concluded that the contamination might have had an even larger negative effect during 2012^34^.

Here, we describe the results of a study on barn swallow nestlings during May-June 2012 to investigate whether exposure to radiation is affecting their genetic integrity prior to fledging. We estimated external radiation exposure of nestlings by attaching thermoluminescent dosimeters (TLDs) to their nest, and by collecting a sample of nest material whose activity concentration we measured using gamma spectrometry in the lab.

We also describe the results of a survey of barn swallows that we conducted in July 2011-2013 across gradients of radioactive contamination spanning almost two orders of magnitude. Part of this database (2011 and 2012) has been previously published in studies relating the abundance of birds in the Fukushima region to the level of radioactive contamination^33^,^34^. In addition to presenting an additional year of data, here we focus the analyses on the local abundance of barn swallows. We also present an analysis of the age ratio of barn swallows, which can be readily determined from plumage characteristics, predicting that higher levels of radiation would lead to a lower fraction of juveniles due to egg infertility and death of nestlings^35^.

With a few notable exceptions^16^,^23^,^24^, all studies conducted so far have at most analyzed the concentrations of radioisotopes in the tissues of organisms, but neglected the assessment of markers of their potential biological effects. The results that we describe represent the first extensive investigation of the potential genotoxicity of measured radiation exposure in any wild population of birds from the Fukushima region.

## Results

### Radioactivity of nest samples and radiation exposure of nestlings

The average exposure measured by the TLDs was 0.59 mGy (0.79 mGy SD; range: 0.15–4.9 mGy; N = 43), corresponding to an average dose rate of 0.90 μ Gy h^−1^ (1.24 μ Gy h^−1^ SD; range: 0.23–7.52 μ Gy h^−1^).

The activity concentrations measured in the nest samples were 10,730 Bq kg^−1^ dry weight (d.w.) (18,276 SD; range 318–82,409 Bq kg^−1^d.w.; N = 45) for ^137^Cs, and 8,656 Bq kg^−1^ d.w. (14,433 SD; range 128–60,940 Bq kg^−1^d.w.; N = 45) for ^134^Cs. When we combined the activities measured for each radionuclide in a single estimate, the total radioactivity was 19,386 Bq kg^−1^ d.w. (32,681 SD; range 478–143,349 Bq kg^−1^d.w.; N = 45). Total radioactivity of the nest material significantly positively predicted the radiation dose received by the TLDs (*t*_39_ = 6.74, *p* < 0.0001, *R^2^* = 0.54, N = 40; Supplementary Fig. 1). Environmental radiation levels significantly positively correlated with the dose received by the TLDs (*t*_42_ = 4.88, *p* < 0.0001, *R^2^* = 0.37, N = 43; Supplementary Fig. 1). Environmental radiation levels also positively correlated with the specific activity of ^137^Cs (*t*_44_ = 2.22, *P* = 0.032, *R^2^* = 0.08, N = 45), and of ^134^Cs (*t*_44_ = 2.37, *P* = 0.022, *R^2^* = 0.09, N = 45).

### Radiation exposure and genetic damage of nestlings

**Figure 1:**
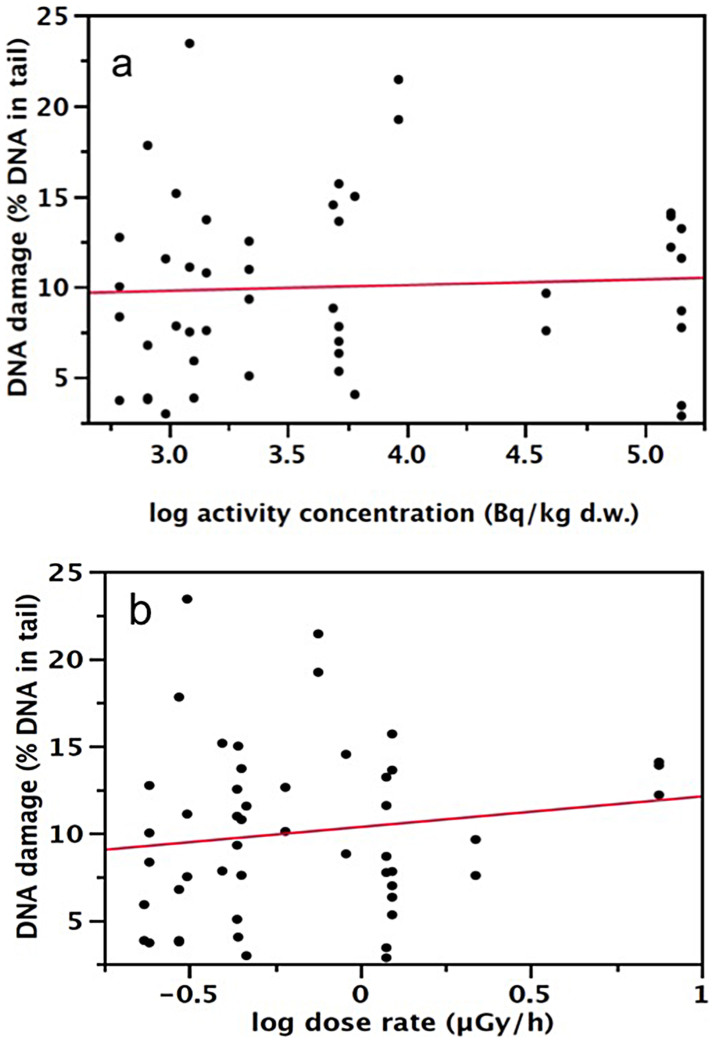
Radiation measurements and genetic damage. The relationship between genetic damage of nestlings and (a) the activity concentrations of the nest material (Bq/kg d.w., summing activities of ^134^Cs and ^137^Cs) or (b) external radiation dose rate, as measured by the TLD (μ Gy h^−1^). The lines are simple regression lines interpolated to the log-transformed data.

The average DNA damage, as indexed by the percentage of DNA in the comet tail, was 10.04 (4.86 SD; range: 2.83–23.41). The total activity concentration of the nest material, obtained by combining the estimates for ^134^Cs and ^137^Cs, did not significantly predict genetic damage of nestlings (*F*_1,6.24_ = 0.51, *p* = 0.502, N = 49, Fig. 1a), nor did the dose to the TLDs attached to the nest (*F*_1,9.81_ = 0.33, *p* = 0.577, N = 49, Fig. 1b). We tested if there was a difference in variance in DNA damage at high radiation levels by splitting our dataset in two groups using median radioactivity of the nest sample or median dose to the TLDs as the cutting points. Variance in DNA damage did not differ significantly between nestlings from more radioactive nests and those from less radioactive nests (Levene's test: *F_1,47_* = 0.015, *p* = 0.904). Similarly, variance in DNA damage did not differ significantly between nests where the TLDs received a higher dose and those where the dose to the TLDs was lower (Levene's test: *F*_1,47_ = 0.039, *p* = 0.844).

In none of these analyses did body mass and estimated age of the nestlings significantly predict genetic damage of the nestlings (see Table 1 in Electronic Supplementary Material). The effect of the nest of origin was never significant in predicting genetic damage of the nestlings (Table 1 in Electronic Supplementary Materials).

### Abundance and age ratio of barn swallows

**Figure 2:**
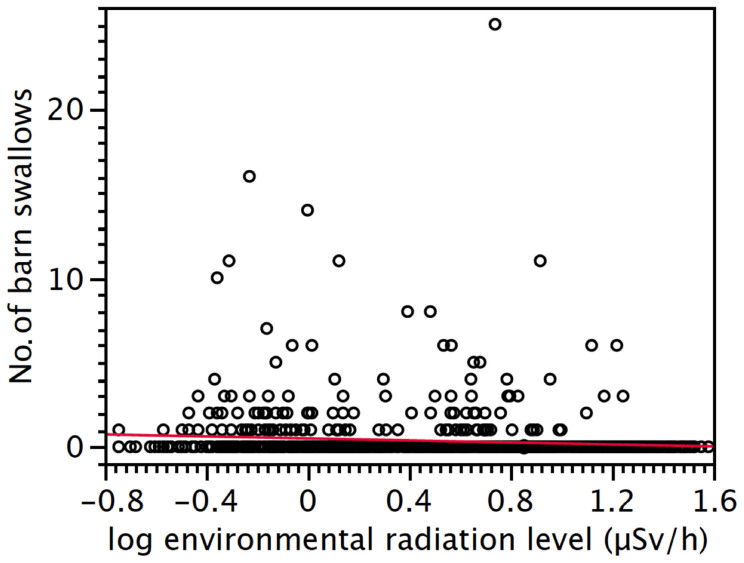
Barn swallows abundance and radioactive contamination. The abundance of barn swallows declined with increasing levels of radioactive contamination as measured during our multi-year point-count census.

Radiation levels at the breeding bird census points ranged from 0.18 to 38.11 μ Sv/h [mean (SD) = 7.16 μ Sv/h (7.90), N = 1100]. The abundance of barn swallows, as inferred from our point count censuses, significantly declined with increasing environmental radiation levels (*F*_1,1093_ = 105.81, *p* < 0.0001; slope (SE) = −1.18 (0.12); Fig. 2). The number of barn swallows increased with increasing farmland (*F*_1,1093_ = 12.53, *p* = 0.0004; slope (SE) = 6.82 × 10^−3^ (1.37 × 10^−3^)), and decreased with increasing ground coverage by grass (*F*_1,1093_ = 320.78, *p* < 0.0001; slope (SE) = −4.75 × 10^−2^ (2.18 × 10^−3^)) and coniferous forest (*F*_1,1093_ = 4.98, *p* = 0.0256; slope (SE) = −1.74 (0.39)). In addition, the abundance of barn swallows differed among years (*F*_2,1093_ = 103.83, *p* < 0.0001). There were significantly fewer barn swallows in 2012 than in 2011 (*t* = 23.1, *p* < 0.0001) and fewer in 2013 compared to 2012 (*t* = 7.85, *p* = 0.005), or the two previous years combined (*t* = 34.07, *p* < 0.0001).

**Figure 3:**
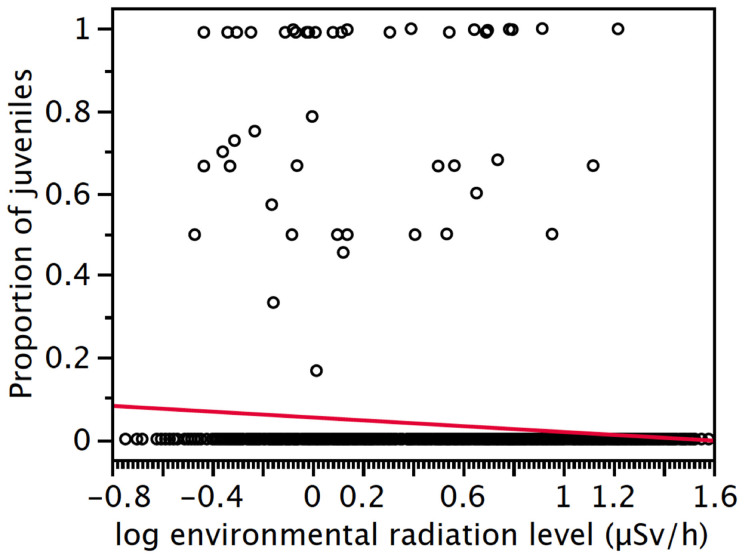
Age ratio of barn swallows and radioactive contamination. The proportion of barn swallows being juveniles declined with increasing levels of radioactive contamination.

The probability of a barn swallow being a juvenile decreased significantly with increasing environmental radiation levels (*F*_1,1069_ = 13.50, *p* = 0.0002; slope (SE) = −1.84 (0.57); Fig. 3). In addition, juvenile barn swallows were more common where adults were more abundant, as expected from the fact that the adults produced the offspring (*F*_1,1069_ = 32.84, *p* < 0.0001; slope (SE) = 1.00 (0.18)). There was also a significant variation among years in the probability that a bird was a juvenile (*F*_2,1069_ = 15.44, *p* = 0.021). This probability was higher in 2012 than in 2011 (*t* = 6.04, *p* = 0.014), or 2013 (*t* = 6.04, *p* = 0.014).

## Discussion

In this study, we investigated genetic damage in barn swallows nestlings exposed to radioactive contamination following the accident at the Fukushima Daiichi Nuclear Power Plant in March 2011. We also estimated the abundance of barn swallows across sites differing in environmental radiation levels by almost two orders of magnitude, while also assessing the relative frequency of juveniles and adults. To the best of our knowledge, this is the first study at Fukushima relating a known biomarker of radiation exposure to estimates of radiation exposure in any wild population of animals exposed to the radioactive fallout.

We could not detect any increase in genetic damage in nestlings exposed to a range of contamination levels during their rearing period. These results partially conflict with previous results in adult barn swallows from the Chernobyl region, where higher genetic damage was demonstrated at levels that were comparable to the ones detected in this study^26^. It could be argued that barn swallow nestlings at Fukushima were exposed for shorter times compared to adults in Chernobyl. The exposure period of barn swallow nestlings (averaging 26 ± 5 days in our sample, due to the combined duration of the incubation period and the rearing period) is considerably shorter compared to the months-long residence of adult barn swallows at the breeding sites. Alternatively, differences in the mixture of radionuclides scattered by the two disasters could explain the difference in the effect, if different mixtures have different associated risks due to differences in particle emission. While ^134^Cs and ^137^Cs are the dominant radionuclides dispersed by the Fukushima disaster^12^, ^137^Cs, ^90^Sr, ^241^Am and several radioisotopes of plutonium are the ones currently present around the Chernobyl Exclusion Zone^36^. The greater abundance of Pu isotopes and other actinides in Chernobyl could thus be responsible for the difference between the two disasters, as alpha emitters have large health effects. Finally, differences in historical exposure and associated trans-generational accumulations of deleterious effects could be responsible for the differences between the two disasters. Swallow populations in the Chernobyl region have been chronically exposed to radioactive contamination for over 20 years at the time of sampling for our 2010 study of genetic damage. Conversely, exposure to radioactive contamination only lasted little more than one year when we sampled barn swallows in the contaminated areas around Fukushima for the present study. While this difference in historical exposure is expected to affect mutation accumulation, predictions regarding the resistance of natural populations to radiation-induced genetic damage are less clear, as genetic damage is not inherited, and natural populations are expected to evolve resistance to radiation-induced oxidative damage over generations^37^. Given that the nestlings examined here belonged to the second generation after the disaster, and the first one from parents that were themselves exposed throughout development, we do not expect the lack of an increase in genetic damage to be due to selection for adaptation to ionizing radiation.

At present, the interpretation of the variation among nestlings in their levels of DNA damage is deemed to be largely speculative. Differences in individual growth rates or in the intensity of competition with siblings could account for such variation through an effect on the oxidative status of nestlings. Consistent with this, resistance of red blood cells to free radicals has been found to negatively correlate with growth rate in zebra finch (*Taeniopygia guttata*) nestlings^38^. In addition, magpie (*Pica pica*) nestlings begging more intensively were shown to have higher levels of lipid peroxidation, as indexed by their malondialdehyde levels^39^. Future studies where nestlings are repeatedly measured will allow controlling for these confounding factors, uncovering potential more subtle effects of radiation.

Due to restriction in access to more highly contaminated areas during our sampling of nestlings, we could not access sites where nestlings might have received considerably higher doses, including the towns of Okuma, Futaba and Namie. Thus, the data presented here should be cautiously interpreted when addressing whether exposure to radioactive contamination is causing an increase in genetic damage in wild populations of animals in contaminated areas, as higher contamination levels might imply more deleterious consequences. The barn swallow is a model species for investigating the effects of radioactive contamination in Fukushima due to its abundance, philopatry to a once chosen breeding site, and availability of control as well as affected populations (e.g. in Chernobyl). However, different species may vary in their radiosensitivity and the lack of an effect in one species does not necessarily imply that all others are similarly unaffected^1^,^2^,^3^,^30^,^31^,^33^,^34^.

In addition, while the biomarker that we assessed did not show any response among nestlings, our census of barn swallows confirmed previous findings of population declines of several bird species in the Fukushima region^33^,^34^. In addition, it suggested that the population decline is due to lower fecundity and/or lower fledging rate, as demonstrated by a decrease in the proportion of juveniles at higher levels of radiation exposure. This result is consistent with the demonstrated decline in fertility, reproductive function and parental care that we have shown in Chernobyl in the barn swallow as well as in other species^35^,^40^,^41^,^42^.

Human absence from highly contaminated towns, with the associated changes in the farming practice and the lack of deterrence for the natural predators of this species (e.g. the Japanese jungle crow *Corvus macrorhynchos*) is a potential alternative explanation for the decline of this species in contaminated areas. In future studies, the assessment of biomarkers of radiation exposure will help determine whether the decline of this species is due to a direct or indirect effect of ionizing radiation (i.e. through an effect on human presence).

The discrepancy between the decline in abundance of barn swallows and the lack of any response in the biomarker of genetic damage that we assessed in barn swallow nestlings calls for further investigation of the potential mechanistic (i.e. physiological and genetic) links between radiation exposure and population dynamics. Multiple cytogenetic biomarkers of radiation exposure will have to be investigated in the future, while at the same time expanding research into areas contaminated to a higher degree than it was assessed in the present study. Similarly, aural ad visual censuses of diversity and abundance will have to be complemented with mist netting of birds in order to estimate transfer of radionuclides to birds. It should be noted that radiation levels examined during the censuses were much wider than the range of contamination levels where nestlings have been measured, as more highly contaminated areas could not be sampled due to lack of access or sampling permit. Thus, the present results should not be interpreted as indicating that no deleterious consequence is expected over the entire area that was contaminated by the radioactive fallout, nor should they be taken as evidence that genetic damage at the *adult* stage is not mediating the population decline of barn swallows, as adult birds were not assessed in the present study. In fact, the investigation of genetic damage of nestlings should also be expanded to more highly contaminated areas in order to exclude the possibility that it contributed to the decline of barn swallow populations.

Overall, our radioactivity measurements are compatible with previously published measurements and dose estimates^43^,^44^. Higher exposure levels for barn swallows can thus be predicted in more highly contaminated areas. The exposure levels measured here are consistent with the occurrence of physiological and life-history consequences (i.e. reduced survival and reproduction) in exposed organisms^44^. A recent analysis that inferred doses from published information on contamination levels and used official benchmarks for dose-response also concluded that exposure to contamination following the accident could induce sub-lethal effects on the populations of terrestrial vertebrates^45^. This same analysis, however, also concluded that any population-level consequence of such individual-level doses would be unlikely, thus raising the issue of reconciling measured doses with population declines that have been shown by recent censuses^33^,^34^. At the same time, it should be noted that our estimates of radiation exposure are conservative, as they admittedly do not account for internal radiation exposure due to inhalation or ingestion of radionuclides. Dose conversion coefficients (DCC) for internal exposure to ^134^Cs and ^137^Cs in species ecologically similar to the barn swallow are expected to be at least as large as the DCCs for external exposure^46^. Thus, future studies will have to improve dosimetry by assessing both internal and external radiation exposure of local populations of barn swallows potentially impacted by the fallout.

Similar population declines at levels of contamination that are not predicted to have population-level consequences have also been observed in the Chernobyl region^31^,^32^,^47^, prompting similar skepticism^48^. There is increasing evidence, however, that the benchmarks indicated as safe by international organizations (IAEA, ICRP)^49^,^50^ might be underestimating the risk associated with exposure to ionizing radiation in the natural environment, especially under chronic exposure^51^. Hazards to natural populations have recently been found to arise at doses considerably lower than it had been shown in controlled experiments in the lab^51^. In addition, a recent meta-analysis that reviewed studies conducted in very high background radiation areas where radionuclides occur naturally found a consistent positive relationship between environmental radiation levels and mutation rate, DNA repair and genetics, in human as well as animal populations^52^. The likely explanation for the discrepancy between the lab and an ecologically-meaningful setting is that lab conditions are far more benign than realistic ecological conditions, where food and essential nutrients are scarcer, predators and parasites are more frequent, and other stressors may make the effects of ionizing radiation more apparent.

## Methods

**Figure 4:**
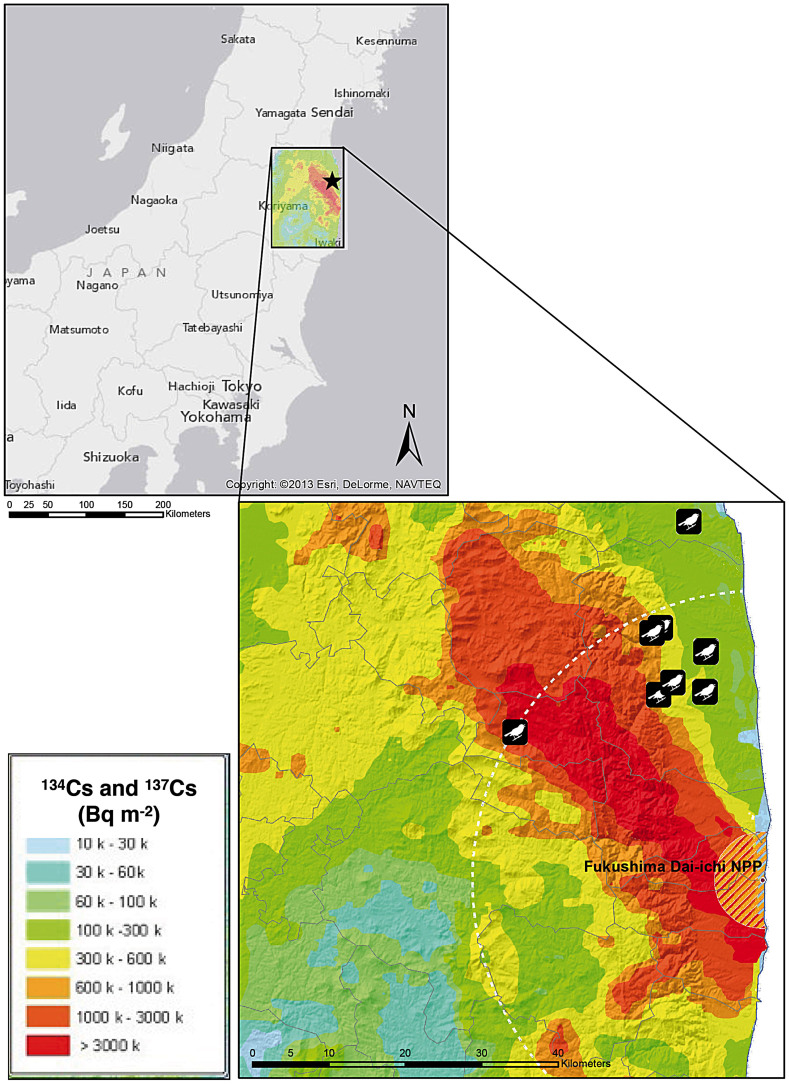
Locations of sampling sites. Locations of the sixteen nests used in the analyses of the relationship between contamination levels and DNA damage of the nestlings. Each location may correspond to more than one nest sampled. Contamination levels are derived from official data from the Japanese Ministry of Education, Culture, Sports, Science and Technology (MEXT), and used to interpolate a map of contamination at 1-m height. The map was created using ArgGis v10.2 (Environmental Systems Research Institute, Redlands, CA). Used with permission. Copyright © 2015 Esri, DeLorme, NAVTEQ. All rights reserved.

During May 2012, we attached thermoluminescent dosimeters (TLDs) to the inner and outer rim of 55 barn swallow nests from the Fukushima region (Fig. 4). We used individually calibrated LiF:Mg,Cu,P TLDs (3.2 × 3.2 × 0.8 mm; GR-200A), which have a higher sensitivity than GR-100 TLDs^53^. The linearity and dose-response of the TLDs were measured with beams produced by a medical linac and a ^137^Cs source. We read the TLD response with a System 310 TLD Reader (Teledyne Brown Engineering), in a temperature range from room temperature to 240°C, at a rate of 10°C/s. The readings were consistent with previously published results^54^. After an average 28.4 days (0.4 SE; range: 25–33 days), we retrieved the TLDs from the nest. On the occasion of retrieving the TLDs, we collected a sample (~1 g) of nest material from the rim of the nest. From 62 chicks from 16 nests that we estimated to be at least 7–8 days old, we also collected a small blood sample (~50 μL), through puncture of the brachial vein and collection in a heparinized capillary tube. We also transferred a drop of blood (~10 μL) to a vial containing RNAprotect (Qiagen).

All procedures were performed in accordance with relevant guidelines and regulations, and approved by the Institutional Animal Care and Use Committee (IACUC) of the University of South Carolina (Protocol number: 2014-100237-052611).

### Radioactivity measurements

In the field, we measured environmental α, β and γ radiation at the ground level below the nest using a hand-held dosimeter (Model: Inspector, SE International, Inc., Summertown, TN, USA).

We measured the activity concentrations of nest samples by conducting gamma ray spectrometry with a SAM 940 Radioisotope Identifier (Berkeley Nucleonics, San Rafael, CA) equipped with a 7.62 × 7.62 cm (3″ × 3″) sodium iodide (NaI) detector. The spectrometer was placed vertically within a lead detector shield (Canberra Industries, Meriden, CT, USA), with additional shielding provided by lead bricks. We measured each sample by placing it on top of the SAM 940 Radioisotope Identifier.

We later converted the spectra to activity measurements after calibration of the instrument using standard ^137^Cs and ^134^Cs sources. For analysis we focused on the 661 keV decay gamma from ^137^Cs and the 597 and 796 keV peaks from ^134^Cs. The samples were dried in a heating oven with mechanical convection (Binder Inc., Bohemia, NY) at the temperature of 60°C for 12 h, and weighed using a Sartorius electronic balance (Model R160P; Göttingen, Germany).

A high statistics, “empty target” spectrum was collected prior to the sample readings and subtracted from all spectra to remove counts not associated with radioactive decay from the sample. A linear background function was then fit to the peak region (490–500 keV) to remove the continuum gammas and isolate the decay peaks. The 597 keV peak from ^134^Cs and the 661 keV peak from ^137^Cs overlapped considerably while the 796 keV peak from ^134^Cs was resolved completely. A spectrum from the ^134^Cs calibration source was normalized to the data spectrum by fitting to the 796 keV peak. This fit was then subtracted from the entire sample spectrum to isolate the 661 keV peak from ^137^Cs. Integrating and comparing the counts in the decay peaks from the samples to the counts in the same peaks from the known calibration sources produced the absolute calibration. The total activity of each sample was calculated by summing the activities estimated for ^137^Cs and ^134^Cs.

To estimate total duration of exposure for each nestling, we summed the estimated age of the nestlings and the duration of the incubation period, which we conservatively estimated at 14 days.

### Analysis of genetic damage

We estimated genetic damage using a single cell gel electrophoresis assay, also known as comet assay, following the protocol reported in Ref. 55, with minor modifications.

We prepared slides in advance by dipping single-frosted slides (VWR, Radnor, PA) in 1.5% normal melting-point agarose. We transferred 3 μL of the solution of blood in RNAProtect (Qiagen) to 997 μL of 1× PBS. We then mixed 50 μL of the solution with 450 μL of 1.5% low melting-point agarose, and layered 100 μL of this mixture on the slides, covering with a glass coverslip. We allowed the agarose to solidify for five minutes at 4°C. We then removed the coverslip and added another layer of 100 μL of low melting-point agarose, and again allowed to solidify for five minutes, before removing the coverslip. The slides were left for 1 hour at 4°C to allow the solidification of the gel, and then immersed in cold lysis buffer (1% sodium sarcosinate, 2.5 M NaCl, 100 mM Na_2_EDTA, 10 mM Tris, 1% Triton X-100 added immediately prior to use, at a final pH = 10), where they were kept for 1 hour at 4°C. We then rinsed the slides with cold ddH_2_O, and immersed them in neutral buffer (300 mM NaOH, 100 mM Tris, pH 10.0), to allow unwinding of the DNA for 30 minutes at 4°C. We electrophoresed slides in a tank filled with the same buffer for 30 minutes at 0.7 V/cm and 150 mA at 4°C. After electrophoresis, we rinsed the slides in a neutralization buffer (0.4 M Tris, pH 7.4) three times, for five minutes each. The slides were then fixed in 70% ethanol for 15 minutes, and left to dry overnight. We ran four slides per each individual.

We stained the slides by immersion in a 1:10,000 solution of SYBR® Gold (Trevigen, Gaithersburg, MD) for five minutes. Slides were then de-stained through immersion in a bath of dd-H_2_O for five minutes, and left to dry. Images of individual cells were captured using a Metafer System (Metasystems, Bethesda, MD), an automated system that performs detection and scoring of individual cells^56^. Only the nestlings for whom we had captured at least 100 cells across the different slides were retained in the final sample^57^. In the final analyses we included 49 nestlings belonging to 16 nests, representing 78% of the initial sample of 63 nestlings. On average we captured 313 cells (147 SD; range: 111–725) from 1–4 slides. As a measure of damage to the DNA we used the percentage of DNA in the tail, which is a measure based on the relative fluorescence intensity of the tail compared to the head of the comet, and the most reliable parameter for the comet assay^57^,^58^.

Data on genetic damage in each nestling were obtained by averaging percentages of DNA in the tail of the comet across all cells.

### Point count censuses

**Figure 5:**
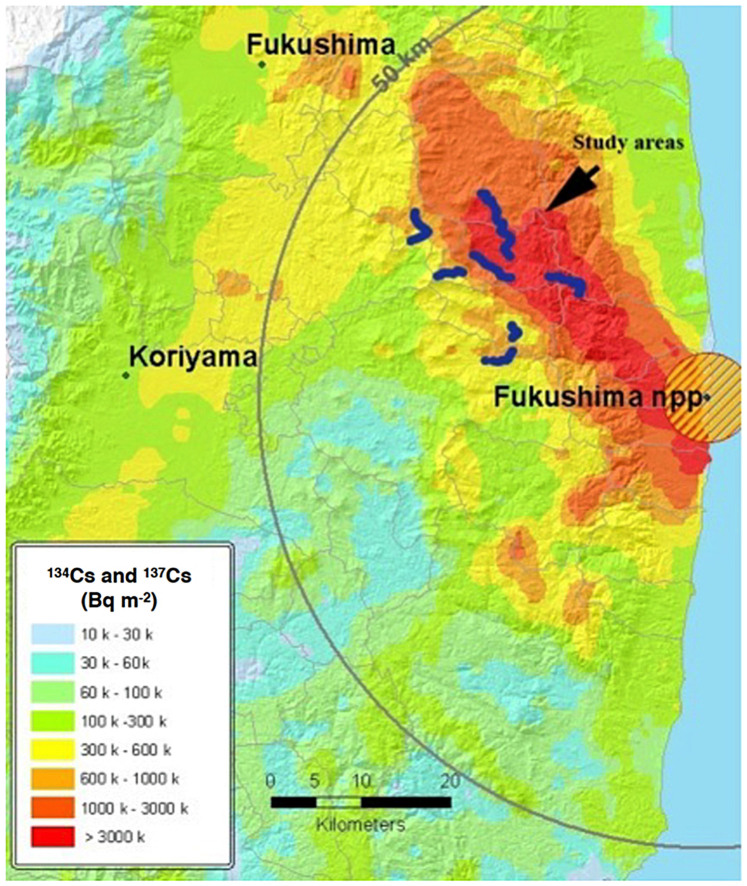
Locations of census sites during 2011–2013. Contamination levels are derived from official data from the Japanese Ministry of Education, Culture, Sports, Science and Technology (MEXT), and used to interpolate a map of contamination at 1-m height. The map was created using ArgGis v10.2 (Environmental Systems Research Institute, Redlands, CA). Used with permission. Copyright © 2015 Esri, DeLorme, NAVTEQ. All rights reserved.

During the first week of July 2011–2013, we conducted a point-count census of birds across clean and contaminated sites (Fig. 5). Each count lasted five minutes, with census points located at approximately 100 m intervals. At each census point, we classified the habitat as being agricultural, grassland, deciduous forest or coniferous forest, and estimated ground coverage by these different habitats (to the nearest 10%) within a distance of 50 m. In total, we collected 1100 5-min point counts (2011: N = 300; 2012: N = 400; 2013: N = 400). The census points were the same in all years, except for 2011, when 100 fewer counts were conducted due to restrictions in access. The relationship between radiation and abundance did not qualitatively change if we restricted the analyses to the 300 points where the counts were conducted in all three years (results not shown). At each census point, we recorded radiation levels using a hand-held dosimeter at ground level (Model: Inspector, SE International, Inc., Summertown, TN, USA). We also recorded the geographic coordinates and altitude (using a GPS), cloud cover at the start of each point count (to the nearest eighth), temperature (degrees Celsius), and wind force (Beaufort). For each census point we recorded time of day at the start of the count (to the nearest minute) and included it in the analyses as an explanatory variable. As activity levels of birds peak in the morning and, to a lesser extent, in the evening, we also included time squared in our analyses. APM conducted all censuses, thus preventing any issue due to inter-observer variability. All the nests that we inspected during 2012–2013 would be fledged by the time we conducted our census. Thus, no difference among years would be expected based on differences in the timing of reproduction.

In a second set of analyses, we analyzed the probability of observing a juvenile barn swallow (as identified from the short tail streamers and the pale coloration, using binoculars) as a function of environmental radiation levels, as well as the same predictors that we included in the analysis of barn swallow abundance. We also included the local abundance of adult barn swallows as a predictor in the analysis, as juvenile barn swallows are the offspring of the adult barn swallows present.

### Statistical analysis

For the analysis of genetic integrity of nestlings we used general linear mixed models (GLMMs) where we included radiation exposure (either log-transformed radioactivity of the nest material or radiation dose as inferred from the TLDs) as a covariate, and the nest of origin as a random effect. In both analyses we included duration of exposure as a covariate. Degrees of freedom were estimated using the Kenward-Roger approximation. All analyses were performed in SAS 9.3 (SAS Inc., Cary, NC).

In the analysis of the abundance of barn swallows, we used generalized linear models, assuming a Poisson distribution of count data. As predictor variables, we included log_10_-transformed radiation and all potentially confounding variables listed above. In addition, we included temperature, cloud cover, wind, time of day and time of day squared, the latter to account for the fact that bird activity has a peak during early morning and a second, milder peak in the afternoon. We also included radiation level squared to account for non-linear relationships between species richness and abundance, respectively, and radiation. These analyses were all implemented in the statistical software JMP (SAS Institute Inc., 2012). In the analyses of the proportion of swallows being juveniles, we relied on general linear models with binomially distributed data and a logit link function.

## Supplementary Material

Supplementary Information Supplementary Information
