# Supplementary material for: Abundance and genetic damage of barn swallows from Fukushima
Source: Sci Rep. 2015 Apr 2;5:9432. doi: 10.1038/srep09432 (PMC5381690; doi:10.1038/srep09432)
Supplement: Supplementary Information [file srep09432-s1.pdf]

Abundance and genetic damage of barn swallows from Fukushima

A. BONISOLI-ALQUATI[1], K. KOYAMA[2], D.J. TEDESCHI[3], W. KITAMURA[4], H. SUKUZI[5], S. OSTERMILLER[1], E. ARAI[6], A. P. MØLLER[7], T. A. MOUSSEAU[1]

[1] *Department of Biological Sciences, University of South Carolina, Columbia, SC 29208, USA,*

[2] *Japan Bird Research Association, Fuchu, Tokyo, Japan*

[3] *Department of Physics and Astronomy, University of South Carolina, Columbia, SC 29208, USA,*

[4] *Faculty of Environmental Studies, Tokyo City University, Yokohama City, Japan,*

[5] *Value Frontier Co., Ltd., Minato, Tokyo, Japan*

[6] *Division of Ecology and Evolutionary Biology, Graduate School of Life Sciences, Tohoku University, Sendai, Japan,*

[7] *Laboratoire d'Ecologie, Systématique et Evolution, CNRS UMR 8079, Université Paris-Sud, Bâtiment 362, F-91405 Orsay Cedex, France*

Correspondence to AB-A:

Tel: (+1) 803 777 8241

Fax: (+1) 803 777 4002

E-mail: andreabonisoli@gmail.com

Running headline:

A. BONISOLI-ALQUATI ET AL. BARN SWALLOWS AFTER FUKUSHIMA

Table 1. GLMMs of DNA damage in barn swallow nestlings as a function of exposure duration, body mass and (a) activity concentration of the nest material (Bq kg^{-1} d.w.) and (b) the dose rate measured by the thermoluminescent dosimeter (TLD) attached to the nest (µGy h^{-1}).

	z	*F*	df	*P*
(a)				
Nest	0.83			0.204
Activity concentration		0.12	1, 6.51	0.735
Exposure duration		0.50	1, 10.3	0.495
Body mass		0.41	1, 9.84	0.535
(b)				
Nest	0.52			0.300
Net dose		1.30	1, 9.01	0.284
Exposure duration		0.03	1, 10.9	0.863
Body mass		1.80	1, 9.03	0.212

Supplementary Figure 1. Radioactivity measurements and radiation exposure. The relationship between environmental radiation levels (measured using a hand-held gamma spectrometer) and (a) the dose rate measured by the TLDs (μGy h^{-1}) ($t_{42} = 4.88, p < 0.0001$, $R^2 = 0.37$, N= 43) and (b) the activity concentration of the nest material (Bq Kg^{-1} d.w.), measured through gamma spectrometry ($t_{43} = 2.43, p = 0.019, R^2 = 0.12$, N= 44). (c) The relationship between activity concentration of the nest material (Bq Kg^{-1} d.w.) and the dose received by the TLDs (μGy h^{-1}) ($t_{39} = 6.74, p < 0.0001, R^2 = 0.54$, N = 40). Data are log-transformed for clarity of presentation. The lines are simple regression lines interpolated to the log-transformed data.

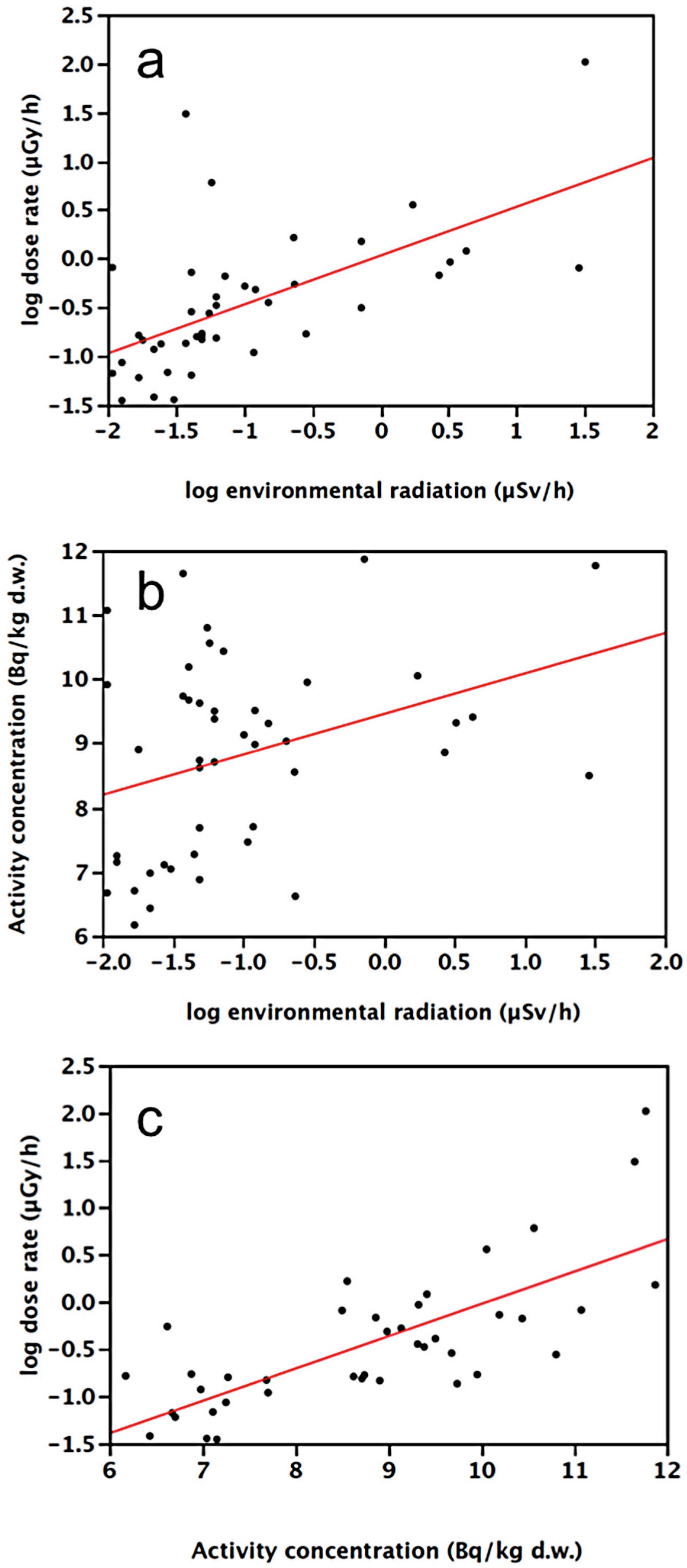
a
2.5
2.0
1.5
1.0
0.5
0.0
−0.5
−1.0
−1.5
log dose rate (μGy/h)
−2
−1.5
−1
−0.5
0
0.5
1
1.5
2
log environmental radiation (μSv/h)
b
12
11
10
9
8
7
6
Activity concentration (Bq/kg d.w.)
−2.0
−1.5
−1.0
−0.5
0.0
0.5
1.0
1.5
2.0
log environmental radiation (μSv/h)
c
2.5
2.0
1.5
1.0
0.5
0.0
−0.5
−1.0
−1.5
log dose rate (μGy/h)
6
7
8
9
10
11
12
Activity concentration (Bq/kg d.w.)
